# Successful treatment of intractable neuralgia in patients with typical and atypical Ramsay Hunt syndrome by transcutaneous facial nerve stimulation: a case series study

**DOI:** 10.3389/fnins.2025.1554490

**Published:** 2025-04-02

**Authors:** Yan-Bo Deng, Ying Zhong, Yu-Xian Lin, Chen Lin, Pin-Pin Ji, Shen-Yin Wang, Wei-Yi Gong

**Affiliations:** ^1^Department of Pain Medicine, Affiliated Hospital of Guilin Medical University, Guilin, China; ^2^Department of Radiology, Affiliated Hospital of Guilin Medical University, Guilin, China

**Keywords:** Ramsay Hunt syndrome, postherpetic neuralgia, otalgia, facial nerve stimulation, neuromodulation

## Abstract

**Objective:**

Typical Ramsay Hunt syndrome (RHS) is a rare peripheral facial neuropathy associated with reactivation of latent varicella-zoster virus in the geniculate ganglion. Atypical RHS is characterized by the involvement of multiple cranial nerves and cervical roots, leading to more complex manifestations. The primary goal of treatment is to reduce the occurrence of late complications, especially in patients with devastating postherpetic neuralgia (PHN). To date, there is no definitive effective treatment. We present a case series of patients with typical and atypical RHS and severe PHN, who were successfully treated with transcutaneous facial nerve stimulation (FNS).

**Materials and methods:**

This is a retrospective case series including two atypical RHS cases and one typical RHS case. The first patient with atypical RHS suffered from persistent otalgia with severe paroxysmal radiation to the dermatome of fifth cranial nerve (CN V) and IX lesion. The second atypical RHS patient with CN V and VII lesions had persistent frontotemporal neuralgia and otalgia, with severe paroxysmal radiation to the CN V and IX dermatome. The third typical patient had persistent otalgia with severe paroxysmal exacerbations. An FNS in the stylomastoid foramen was successfully performed under ultrasound guidance in combination with DSA. Pain assessment was performed during treatment and follow-up, including the type of pain (persistent pain, breakthrough pain, and tactile allodynia) and pain distribution. Pain intensity was assessed using the Number Rate Scale (NRS) and the Verbal Rating Scale (VRS). The therapeutic effect was assessed using the Pain Relief Scale (PRS). In addition, the Pain Relief Ratio (PRR) was calculated as (NRS_Pre_-T - NRS_Post_-T)/NRS_Pre_-T × 100%, and the treatment was considered effective if the PRR was greater than 50%.

**Results:**

The t-FNS showed excellent pain relief, particularly for breakthrough pain. The breakthrough pain completely ceased before the FNS was turned off, and the persistent pain decreased from moderate to mild intensity before the patients were discharged. The mild persistent pain of the first patient on the 3-month follow-up and the third patient on the 2-month follow-up had completely disappeared, but the mild persistent pain of the second patient was still felt in the temporal region for 1 year.

**Conclusion:**

For the first time, transcutaneous FNS was successfully used to treat intractable PHN in patients with typical and atypical RHS. However, further research is needed to determine the optimal procedure and specific stimulation parameters.

## Introduction

Ramsay Hunt syndrome (RHS), with an incidence of approximately 5 per 100,000 people per year (Murakami et al., 1997; Tiemstra and Khatkhate, 2007), is caused by the reactivation of the herpes zoster virus in the geniculate ganglion. Therefore, the prophylactic use of a vaccine against the herpes zoster virus in high-risk patients can reduce the incidence of RHS. Typical RHS is characterized by a combination of three symptoms: painful vesicles, otalgia, and ipsilateral facial palsy. However, there are some unusual variants associated with atypical RHS (Nishizawa et al., 2021; Zhang and Wei, 2020; Lee et al., 2014). Although herpes zoster is inherently a self-limiting disease, herpetic neuralgia is the first symptom that can appear without facial paralysis or rash (Coulson et al., 2011). Moreover, postherpetic neuralgia, a devastating complication of RHS, lasts longer than the healing of the rash (Choo et al., 1997). Patients often report a decreased quality of life and difficulties with daily activities, which can affect their physical, psychological, and social well-being. Therefore, the primary goal of treatment is to reduce the incidence of postherpetic neuralgia and to achieve better pain relief.

Currently, there are individual case reports on the treatment of RHS. However, early diagnosis and intervention within 72 h of symptom onset are crucial. Effective pain relief often requires a multimodal approach that includes medication (NSAIDs, opioids, tricyclic antidepressants, and anticonvulsants) (Liao et al., 2021), nerve block (Jacques et al., 2019; Liao et al., 2021), acupuncture (Giralt et al., 2020; Liao et al., 2021), and physical therapy (linear polarized near-infrared light irradiation) (Liao et al., 2021). Jeon and Lee (2018) recommended using a combination of antiviral agents (e.g., acyclovir) and corticosteroids to manage RHS-related neuralgia, particularly when administered early to mitigate nerve damage and improve outcomes (Jeon and Lee, 2018). Jacques et al. (2019) also presented a case in which blockade of the terminal branches of the nervus intermedius successfully reduced neuralgia symptoms for 3 months in a patient with severe pain due to Ramsay Hunt syndrome (Jacques et al., 2019). Subdermal injections of BoNT have been shown to be potentially beneficial in cases of RHS with severe ear lobes (Soumekh, 2024), and pulsed radiofrequency (PRF) applied to the greater auricular nerve has provided significant pain relief in refractory otalgia following RHS (Kim et al., 2021). Liao et al. (2021) implemented a multimodal approach, combining oral gabapentin, pulsed radiofrequency (PRF) application to the Gasserian ganglion for pain in the trigeminal nerve region, linear-polarized near-infrared light irradiation for pain in the facial nerve region, and 2% lidocaine spray for pain in the glossopharyngeal nerve region. This method improved pain management and quality of life in a 78-year-old patient with a 3-month history of PHN secondary to RHS with polycranial nerve (V, VII, VIII, and IX) involvement (Liao et al., 2021). Tympanic nerve neurectomy resulted in significant pain relief and improvement in quality of life in a 45-year-old patient with chronic pain in the right-sided facial, ear, and jaw that persisted for 9 years after RHS (Sarathy et al., 2023). However, these techniques have only proven their effectiveness in individual cases.

In this study, we present two patients with atypical RHS who experienced severe neuralgia in unusually extensive facial and cervical dermatomes without facial paralysis. Additionally, we included a typical RHS patient with severe otalgia and ipsilateral facial paralysis after recovery from herpes. The intractable neuralgia was successfully treated with transcutaneous facial nerve stimulation (t-FNS). This study aims to evaluate the safety and efficacy of ultrasound-and DSA-guided t-FNS in patients with refractory RHS neuralgia, addressing the limitations of existing treatments and exploring its potential as an alternative neuromodulation therapy.

### Materials and methods

Patients were selected from the Affiliated Hospital of Guilin Medical University based on a diagnosis of refractory RHS neuralgia. The inclusion criteria included (1) confirmed diagnosis of RHS neuralgia with or without other cranial nerves involved, (2) no response to conventional treatments, and (3) eligibility for neurostimulation therapy. The exclusion criteria included (1) contraindications to neurostimulation and (2) severe comorbidities that could affect the treatment response. The retrospective analysis involved three patients with RHS who suffered from severe PHN and were successfully treated with FNS. Before implanting the t-FNS lead, a Nadbath–Rehman block was performed at the stylomastoid foramen with 1 mL of 1% lidocaine immediately after breakthrough pain recurred, and the pain stopped immediately. FNS at the stylomastoid foramen was then recommended. We discussed the risks of the procedure with the patient, which included bleeding, nerve injury, and infection, along with the potential benefits and alternatives. Once all questions were answered and the patient understood and accepted the procedure, informed consent was obtained.

### Implantation of transcutaneous FNS and parameter program

**Figure 1:**
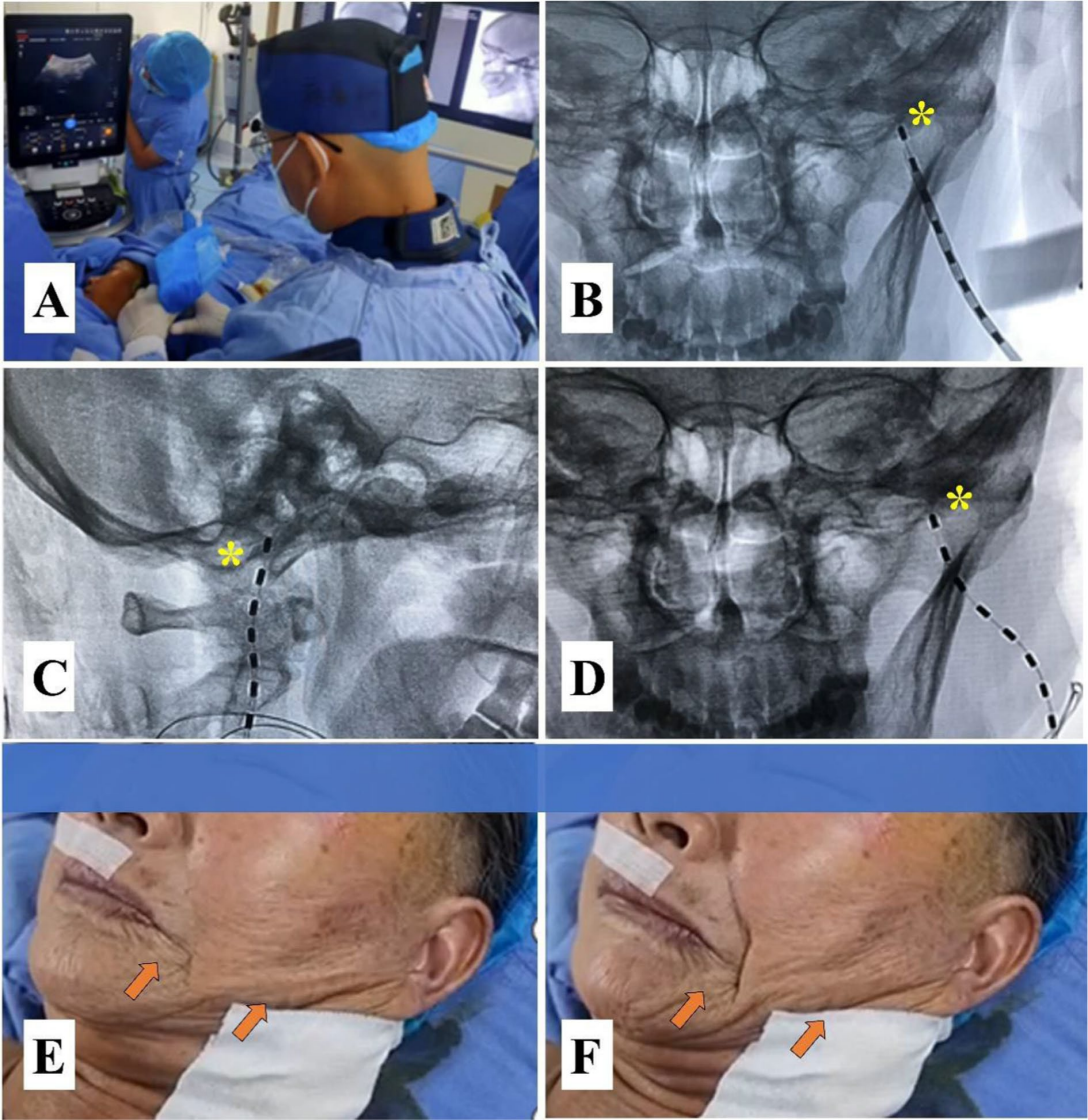
A pilot protocol for transcutaneous facial nerve stimulation (t-FNS). **(A)** Ultrasound-guided injection of 0.5% lidocaine for regional anesthesia and widening of the puncture route to avoid intravascular injection. **(B)** The lead was implanted medial to the MP using a manual 15° arcuate hollow needle in the anteroposterior view of the DSA. **(C)** The lead was again placed anterior to the MP in the lateral view of the DSA. **(D)** The anteroposterior view of the DSA confirmed that the lead was placed medial to the MP. **(E)** A slight contraction of the facial muscle was elicited by a test stimulation voltage of 0.3 V. **(F)** A significant contraction of the facial muscle was elicited by a stimulation voltage of 1 V. The yellow asterisk indicates the MP, and the orange arrow represents the contraction of the facial muscle.

As no technical procedure was available, a pilot protocol for t-FNS was developed, and the procedure was performed under ultrasound guidance in combination with DSA (Figure 1).

#### Anesthesia

The patient was positioned supine on a DSA table with the head tilted to the opposite side. Blood pressure, heart rate, oxygen saturation, and electrocardiographic waveforms were continuously monitored. She received oxygen at 3 L/min and a continuous intravenous infusion of 0.9% saline. Midazolam (Jiangsu Enhua Pharmaceutical Co. Ltd.) at a dosage of 1 mg and fentanyl (Jiangsu Enhua Pharmaceutical Co. Ltd.) at 50 μg were injected intravenously for sedation. For regional anesthesia, 10 mL of 0.5% lidocaine was injected into the puncture site tissue under ultrasound guidance using an 8–3 MHz transducer (Sonosite Inc., Bothell, USA).

#### Implantation

A manual 15° arcuate hollow needle (16 G) connected to a 5-ml syringe with continuous negative pressure was inserted into the skin and slowly advanced toward the foramen stylomastoideum. The procedure was guided by ultrasound to avoid vascular injury, and DSA confirmed the target at the medial and anterior parts of the mastoid process. As soon as the patient complained of discomfort or blood flowed into the negative pressure syringe, the puncture procedure had to be stopped. After the direction was adjusted, the procedure was continued until the needle reached the target. A trial lead (3,086, Abbott Medical, Plano, USA) was implanted through the insertion needle until it touched the bone. Then, the introducer needle was withdrawn, and the placement of the lead was reconfirmed by DSA at the medial and anterior parts of the mastoid process (MP).

#### Testing

The placement of the lead was confirmed by a stimulation test after the lead was connected to the external stimulator (3,510, St. Jude Medical, Inc. Fullerton, CA). The lead was placed close to the facial nerve when the contraction of the facial muscle was evoked by a voltage of less than 0.5 V. The lead was anchored into the skin using sterile 3.0 sutures (Vicryl, Ethicon, Peterborough, ON), and a neck collar was worn to prevent displacement of the lead.

#### Parameter settings and treatment plan

The contact polarity was set as follows: Contact point 1 (−) and contact point 2 (+); the stimulation frequency was 40 Hz, and the pulse width was 200 μs. The amplitude adjustment from 0.5 V to 1 V allowed contraction of the facial muscles that did not disturb the patient’s sleep, and the amplitude adjustment from 1 V to 3 V resulted in a stronger contraction of the facial muscles up to the patient’s upper tolerance limit during the non-sleep period.

### Pain assessment

Depending on the type of pain, including persistent pain (tingling, burning, and itching), breakthrough pain (stabbing and shooting), and tactile allodynia, the patient reported the pain distribution as “yes” or “no” and rated the pain intensity using the NRS (‘0’ for no pain and ‘10’ for unbearable pain) and the Verbal Rating Scale (VRS) (0 = no pain; 1 = mild pain; 2 = moderate pain; and 3 = severe pain or 4 = intense pain). The patient also rated the efficacy using the Pain Relief Scale (PRS) (0 = none, 1 = mild, 2 = moderate, 3 = severe, and 4 = total). In addition, the Pain Relief Ratio (PRR) was calculated as (NRSPre-T - NRSPost-T)/NRSPre-T × 100%, and treatment was considered effective if the PRR was greater than 50%.

## Results

The FNS showed excellent pain relief in all three cases, especially for breakthrough pain. The details are described in detail below.

### Case one: atypical RHS

A 71-year-old female patient suffering from a rash in the left external auditory canal was diagnosed with herpes zoster 15 days ago and received intravenous acyclovir (0.25 g three times daily) for 7 days. She also reported pain in the area of the rash and was given oral gabapentin (initial dose 300 mg three times daily and up to 600 mg three times daily) and tramadol extended-release capsules (100 mg daily). However, her pain worsened over time, and she was referred to our hospital for further treatment.

**Figure 2:**
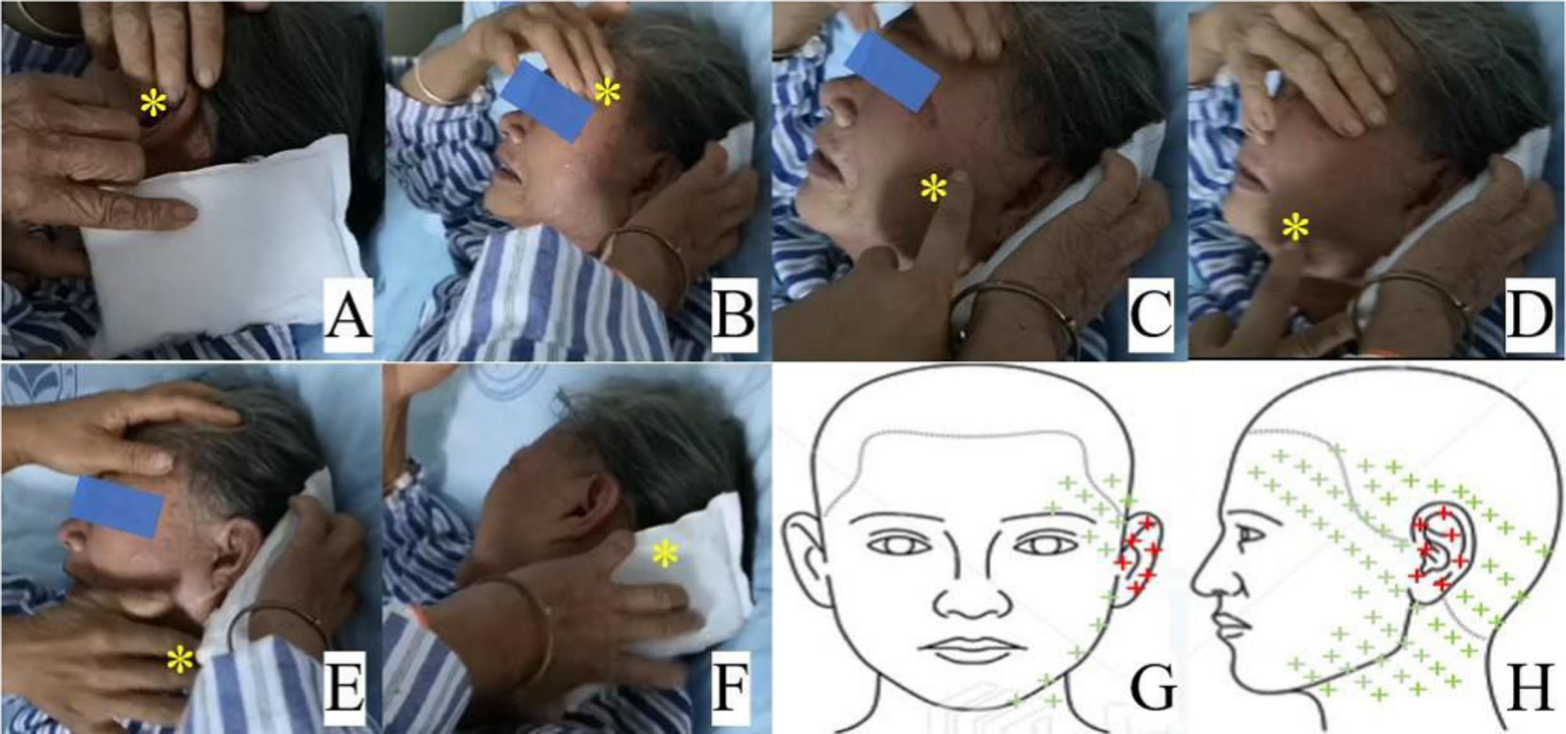
Pain regions of the breakthrough pain attack **(A–F)** and the pain distribution in the schematic representation **(G,H)**. A: external auditory canal and auricle; **(B)** frontotemporal region; **(C,D)** mandibular region; **(E)** pharyngeal and upper cervical region; **(F)** occipital region; **(G,H)**: schematic diagram showing pain distribution of persistent pain in red plus and breakthrough pain in red and green plus. The yellow asterisk indicates the region of breakthrough pain.

The patient complained of a persistent type of pain with paroxysmal exacerbations in intensity. The persistent pain was “incessant tingling, burning, and itching” in the external auditory canal and auricle, with an intensity of 5 on a numerical rating scale (NRS) of 0–10. She also reported a severe, stabbing, and shooting otalgia that occurred almost half an hour apart, lasted for 3–5 min, and radiated to the frontotemporal, mandibular, pharyngeal, upper cervical, and occipital regions with an intensity of 10 on an NRS (Figure 2). The patient was unable to fall asleep and expressed exaggerated fear, anxiety, and depression. Physical examination revealed that tactile allodynia can be triggered when the external auditory canal is lightly touched with a cotton swab, and the cervico-occipital region needs to be cooled when the breakthrough pain occurs.

**Figure 3:**
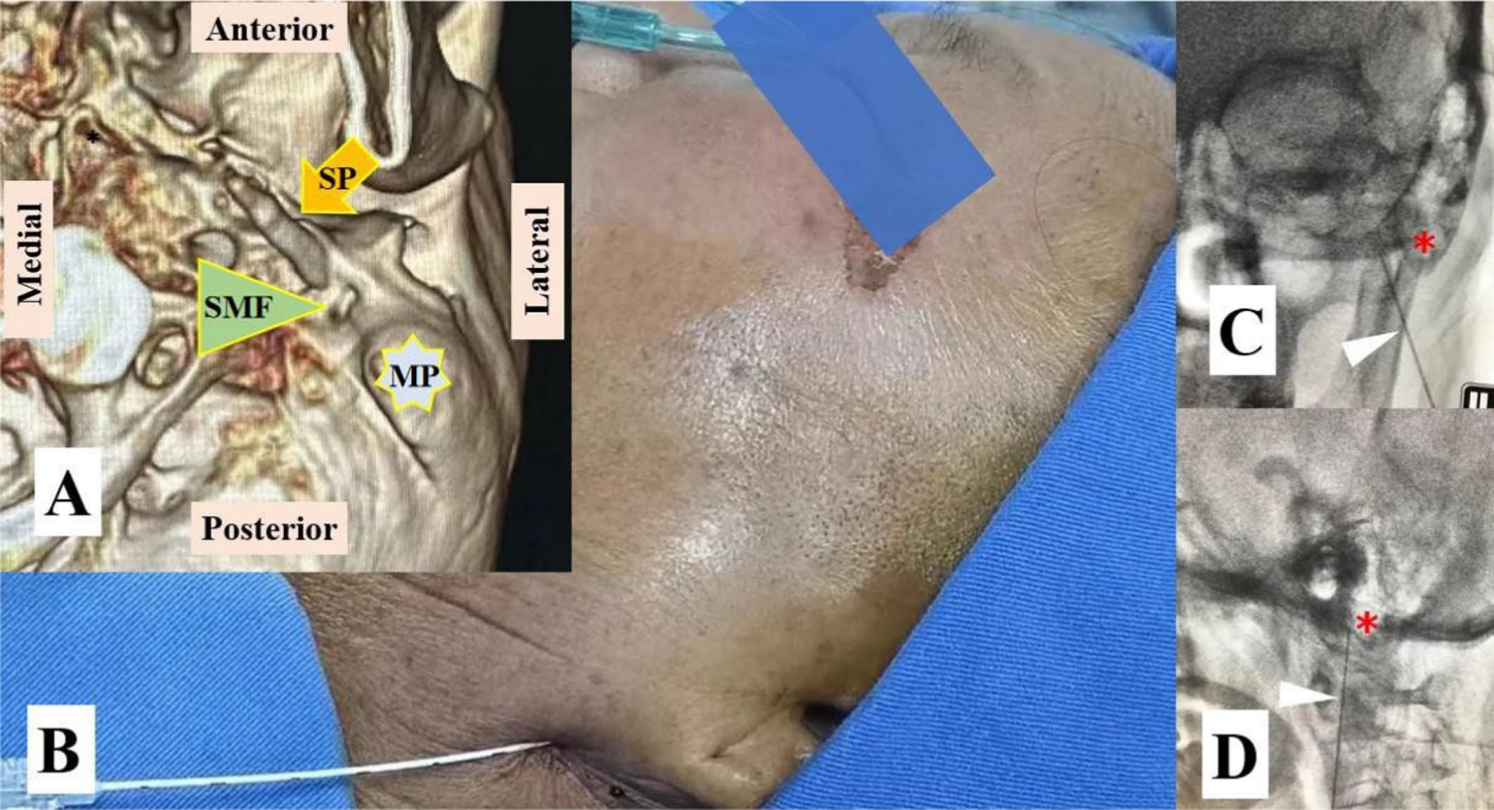
Pulsed radiofrequency (PRF) at the stylomastoid foramen of the facial nerve. **(A)** VR 3D image showing the stylomastoid foramen (SMF) between the mastoid process (MP) and the styloid process (SP). **(B)** PRF was performed with a modified procedure based on the Nadbath– Rehman block method; **(C)** The RF needle is located medial to the MP in the anteroposterior view of digital subtraction angiography (DSA); **(D)** The RF needle is located anterior to the MP in the lateral view of DSA. The red asterisk represents the MP and the white triangle represents the RF needle.

After ruling out the conditions, such as tumors, hemorrhage, swelling, infection, or inflammation through magnetic resonance imaging of the brain and cervical spine, a diagnostic nerve blockade of the supraorbital nerve, glossopharyngeal nerve, greater occipital nerve, lesser occipital nerve, and cervical 2 (C2) nerve with 1% lidocaine was performed based on the affected painful dermatome; however, it was unsuccessful. When the breakthrough pain recurred, a Nadbath–Rehman block was performed at the stylomastoid foramen using 1 mL of 1% lidocaine, and the pain stopped immediately. Following the result, she was diagnosed with atypical RHS with severe otalgia radiating to the dermatome of the trigeminal, glossopharyngeal, and occipital nerves, with no facial paralysis. The patient was then recommended pulsed radiofrequency (PRF) at the stylomastoid foramen. After obtaining informed consent, PRF of the facial nerve (90 V for 10 min each, twice) was performed under the guidance of digital subtraction angiography (DSA) (Figure 3). However, the patient only experienced less than 20% pain relief for approximately 4 h. Then, t-FNS at the stylomastoid foramen was recommended to the patient based on our successful experience treating postherpetic neuralgia with spinal cord and trigeminal nerve stimulation.

The patient herself evaluated the clinical efficacy before and after treatment. Persistent pain was rated as moderate before treatment. The pain decreased by 60% on the fifth day of treatment and had disappeared after 3 months. The range of persistent pain was reduced from the external auditory canal and auricle before treatment to the external auditory canal on the fourth day of treatment until it disappeared during the 3-month follow-up. FNS showed an excellent effect on breakthrough pain. Her breakthrough pain decreased by more than 50% on day one and disappeared completely on day five. The frequency of breakthrough pain decreased dramatically from more than 24 times daily before treatment to four times on the first day, two times on the second day, one time on the third day, and two times on the fourth day during treatment, and it disappeared on the fifth day. The duration of each seizure decreased from 3–5 min before treatment to 0.5–1 min on the first day, 30 s on the second day, 20 s on the third day, and 5 s on the fourth day during treatment. The radiating pain to the frontal and temporal regions on the first day, to the mandibular and pharyngeal regions on the second day, and the occipital region and auricle on the third day had completely disappeared, and the breakthrough pain occurred only in the external auditory meatus (EAM) on the fourth day. Tactile allodynia disappeared during the 3-month follow-up. The patient reported stable efficacy when the t-FNS was switched off on day 7, with no relapses (Table 1).

**Table 1:** The efficacy of transcutaneous facial nerve stimulation (t-FNS).

Nature	Standard		Pre-T	Post-T													
				1D	2D	3D	4D	5D	6D	7D	8D	1 M	2 M	3 M	6 M	9 M	1Y
Persistent Pain	Location (Y/N)	EAM	Y	Y	Y	Y	Y	Y	Y	Y	Y	Y	Y	N	N	N	N
		auricle	Y	Y	Y	Y	N	N	N	N	N	N	N	N	N	N	N
	VRS		2	2	2	2	1	1	1	1	1	1	1	0	0	0	0
	PRS			0	1	1	1	2	3	3	3	3	3	4	4	4	4
	NRS		5	4	3	3	3	2	2	1	1	1	1	0	0	0	0
	PRR (%)			20	40	40	40	60	60	80	80	80	80	100	100	100	100
Breakthrough Pain	Location (Y/N)	EAM	Y	Y	Y	Y	Y	N	N	N	N	N	N	N	N	N	N
		auricle	Y	Y	Y	N	N	N	N	N	N	N	N	N	N	N	N
		FT	Y	N	N	N	N	N	N	N	N	N	N	N	N	N	N
		mandibular	Y	Y	N	N	N	N	N	N	N	N	N	N	N	N	N
		pharyngeal	Y	Y	N	N	N	N	N	N	N	N	N	N	N	N	N
		Upper cervical	Y	Y	N	N	N	N	N	N	N	N	N	N	N	N	N
		Occipital	Y	Y	Y	N	N	N	N	N	N	N	N	N	N	N	N
	F(per/day)		>24	4	2	1	2	0	0	0	0	0	0	0	0	0	0
	Duration		3-5 min	0.5-1 min	30Sec	20 Sec	5 Sec										
	VRS		4	3	2	2	2	0	0	0	0	0	0	0	0	0	0
	PRS			3	3	3	3	4	4	4	4	4	4	4	4	4	4
	NRS		10	5	4	4	4	0	0	0	0	0	0	0	0	0	0
	PRR (%)			50	60	60	60	100	100	100	100	100	100	100	100	100	100
Allodynia	Y/N		Y	Y	Y	Y	Y	Y	Y	Y	Y	Y	Y	N	N	N	N

Patients reported their pain intensity using the Numerical Rating Scale (NRS) (“0” for no pain and “10” for unbearable pain) and the Verbal Rating Scale (VRS) (no pain = 0; mild pain = 1; moderate pain = 2; and severe = 3, intense pain = 4). Patients rate the treatment using the Pain Relief Scale (PRS) (none = 0, a little = 1, some = 2, a lot = 3, complete = 4). In addition, the Pain Relief Ratio (PRR) was calculated as (NRS _pre-treatment_ - NRS _post-treatment_)/NRS _pre-treatment_×100%. Pre-T, Pre-treatment; Post-T, Post-treatment; F, Frequency; EAM, external auditory meatus; FT, frontal-temporal region; Y, Yes; N, NO.

### Case two: atypical RHS with CN V involvement

A 55-year-old male patient presented to our pain clinic with severe neuralgia in the left head and face. He had a rash in the external auditory canal and frontotemporal region 1 month before his admission to our clinic. He was given intravenous acyclovir (0.25 g three times daily) for herpes simplex virus and oral gabapentin (initially 300 mg three times daily and up to 600 mg three times daily) and tramadol extended-release capsules (100 mg daily) for pain relief. However, his aggravated pain made him unbearable after the rash improved; in particular, he was overwhelmed by the breakout pain.

**Figure 4:**
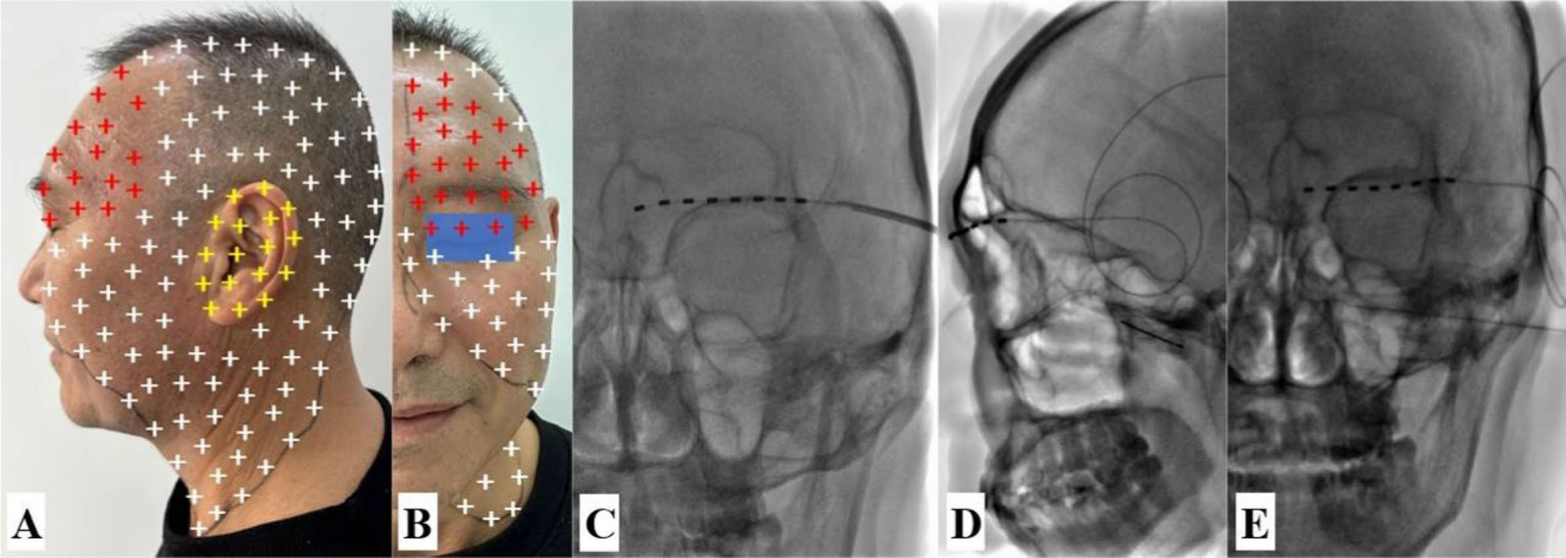
Schematic representation of pain distribution, transcutaneous supraorbital nerve stimulation (t-SNS), and diagnostic maxillary nerve block. **(A,B)** Persistent pain region (red and yellow plus) and eruptive pain region (red, yellow and white plus). **(C)** The t-SNS was implanted under DSA guidance. **(D,E)** DSA-guided diagnostic maxillary nerve block via the pterygopalatine fossa was confirmed in the lateral and anteroposterior views. The white arrow points to the needle.

The patient also reported a persistent type of pain with paroxysmal exacerbations in intensity. His persistent pain was “incessant tingling, burning, and itching” in the external auditory canal, auricle, and frontal-temporal regions with an intensity of 5/10 on an NRS. His severe stabbing and shooting otalgia and frontal-temporal neuralgia radiated to the infraorbital, mandibular, pharyngeal, upper cervical, and occipital regions less than an hour apart, and the intensity was 10/10 on an NRS and lasted approximately 5–6 min during the pain attack (Figures 4,B). The patient could not fall asleep and expressed exaggerated fear, anxiety, and depression. Physical examination revealed pigmentation and hypoesthesia of the skin in the supraorbital and frontal regions and exhibited tactile allodynia of the skin in the supraorbital frontal-temporal region and external auditory canal.

**Figure 5:**
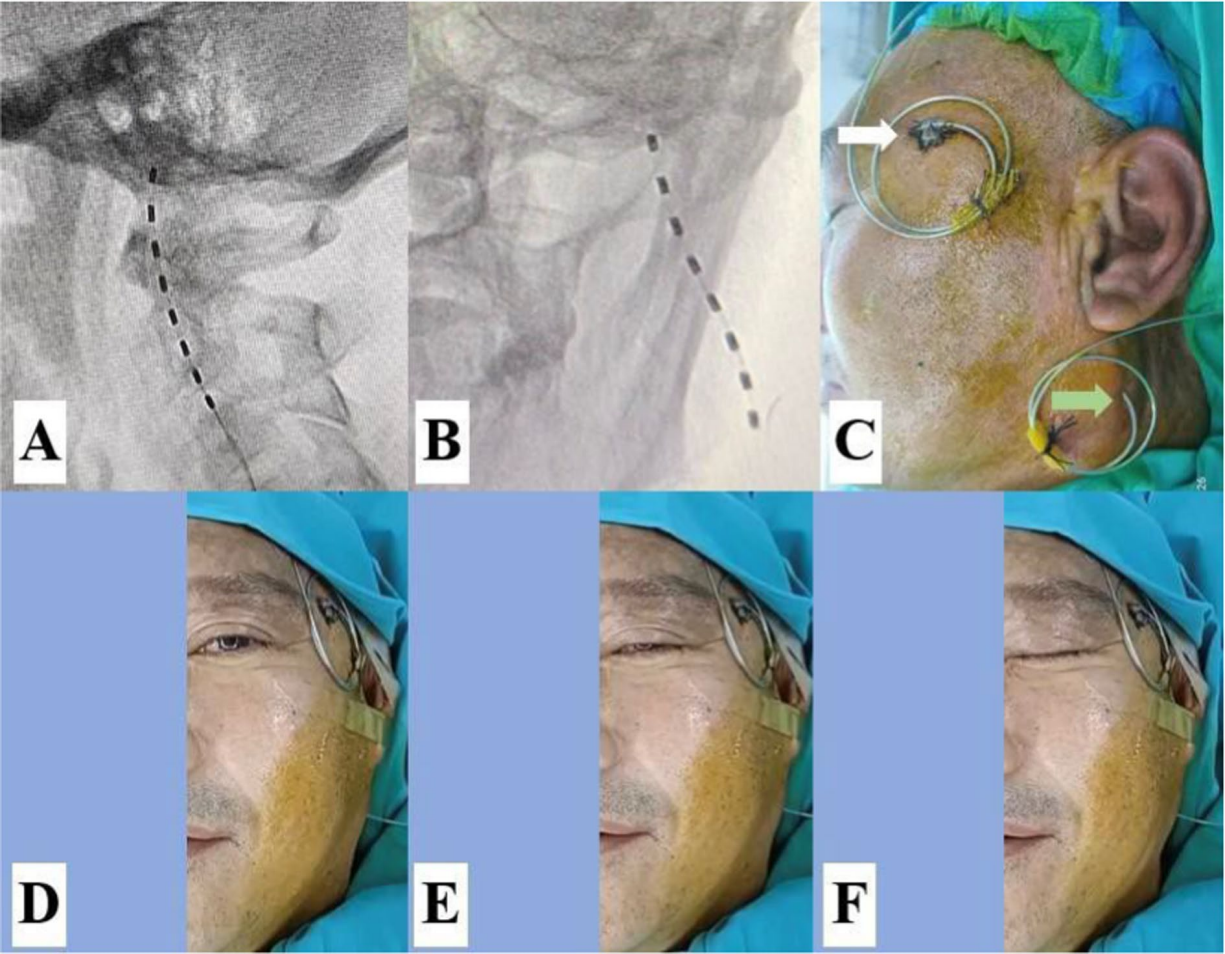
It was confirmed that the lead was placed close to the facial nerve at the stylomastoid foramen. **(A,B)** The lead tip was located anterior and medial to the MP. **(C)** The lead for transcutaneous supraorbital nerve stimulation (t-SNS, white arrow) and the lead for transcutaneous facial nerve stimulation (t-FNS, green arrow) were attached separately to the skin. **(D)** Slight ptosis was observed before the stimulation test. **(E,F)** The slight and distinct contractions of the facial muscles were elicited by the stimulation test at 0.3 V and 0.5 V.

Based on the affected painful dermatome, the pain in the frontal and temporal areas decreased significantly after the blockade of the supraorbital nerve with 1% lidocaine. Transcutaneous supraorbital nerve stimulation was performed after informed consent was obtained, and his persistent pain and breakthrough pain in the frontal and temporal regions also decreased, but there was no benefit in other regions (Figure 4C). Diagnostic blockade of the maxillary nerve was completely ineffective (Figures 4D,E), but his severe paroxysmal pain stopped immediately after diagnostic facial nerve block at the stylomastoid foramen with 1 mL of 1% lidocaine. Based on the symptoms and physical signs in conjunction with the results of the diagnostic nerve block, the patient was diagnosed with atypical RHS involving the fifth cranial nerve (CN V). The t-FNS was recommended based on our previous successful treatment of case 1, and the risks, benefits, and alternatives of the procedure were discussed with the patient. After obtaining informed consent, the procedure was successfully performed according to the pilot protocol (Figure 5).

t-FNS combined with t-SNS showed excellent pain relief in atypical RHS with CN V involvement. The moderate intensity of persistent pain decreased by more than 50% on day 9. On day 11, persistent pain in the forehead region and auricle had completely disappeared, and on day 15, after treatment, the pain in the external auditory canal completely disappeared. However, his mild, persistent pain in the temporal region persisted for a year. Before the treatment, the patient suffered from severe paroxysmal pain. The treatment resulted in excellent pain relief, including intensity, frequency, duration, and distribution of pain during paroxysmal exacerbations. The intensity, frequency, and duration of the pain attacks were already reduced by 50% on the first day and ceased completely on day 15 after treatment. In addition, the treatment stopped the onset in the pharyngeal and upper cervical regions on the third day, in the infraorbital and mandibular regions on the fifth day, in the frontal and occipital regions on the seventh day, and in the auricle on the thirteenth day. Finally, the idiopathic otalgia completely disappeared on day 15. However, his mild tactile allodynia in the temporal region was still present for 1 year (Table 2).

**Table 2:** The efficacy of transcutaneous facial nerve stimulation (t-FNS) and supraorbital nerve stimulation (t-SNS).

Nature	Standard		Pre-T	Post-T													
				1D	3D	5D	7D	9D	11D	13D	15D	1 M	2 M	3 M	6 M	9 M	1Y
Persistent Pain	Location (Y/N)	Frontal	Y	Y	Y	Y	Y	Y	N	N	N	N	N	N	N	N	N
		Temporal	Y	Y	Y	Y	Y	Y	Y	Y	Y	Y	Y	Y	Y	Y	Y
		EAM	Y	Y	Y	Y	Y	Y	Y	Y	N	N	N	N	N	N	N
		auricle	Y	Y	Y	Y	Y	Y	N	N	N	N	N	N	N	N	N
	VRS		2	2	2	2	1	1	1	1	1	1	1	1	1	1	1
	PRS			0	1	1	1	2	3	3	3	3	3	3	3	3	3
	NRS		5	3	3	3	3	2	2	1	1	1	1	1	1	1	1
	PRR (%)			40	40	40	40	60	60	80	80	80	80	80	80	80	80
Breakthrough Pain	Location (Y/N)	Frontal	Y	Y	Y	Y	N	N	N	N	N	N	N	N	N	N	N
		Temporal	Y	Y	Y	Y	Y	N	N	N	N	N	N	N	N	N	N
		EAM	Y	Y	Y	Y	Y	Y	Y	Y	N	N	N	N	N	N	N
		auricle	Y	Y	Y	Y	Y	Y	Y	N	N	N	N	N	N	N	N
		infraorbital	Y	Y	Y	N	N	N	N	N	N	N	N	N	N	N	N
		mandibular	Y	Y	Y	N	N	N	N	N	N	N	N	N	N	N	N
		pharyngeal	Y	Y	N	N	N	N	N	N	N	N	N	N	N	N	N
		Upper cervical	Y	Y	N	N	N	N	N	N	N	N	N	N	N	N	N
		Occipital	Y	Y	N	Y	N	N	N	N	N	N	N	N	N	N	N
	F (per-day)		>24	4	8	5	3	2	3	1	0	0	0	0	0	0	0
	Duration		5-6 min	2-3 min	2 min	10 Sec	10 Sec	5 Sec	5 Sec	2 Sec							
	VRS		4	2	2	2	2	1	1	1	0	0	0	0	0	0	0
	PRS			2	2	2	2	3	3	3	4	4	4	4	4	4	4
	NRS		10	4	4	4	4	3	2	2	0	0	0	0	0	0	0
	PRR (%)			60	60	60	60	70	80	80	100	100	100	100	100	100	100
Allodynia	Y/N		Y	Y	Y	Y	Y	Y	Y	Y	Y	Y	Y	Y	Y	Y	Y

Patients reported their pain intensity using the Numerical Rating Scale (NRS) (‘0’ means no pain and ‘10’ means unbearable pain) and the Verbal Rating Scale (VRS) (no pain = 0; mild pain = 1; moderate pain = 2; and severe = 3, intense pain = 4). Patients rate the treatment using the Pain Relief Scale (PRS) (none = 0, a little = 1, some = 2, a lot = 3, complete = 4). In addition, the Pain Relief Ratio (PRR) was calculated as (NRS _Pre-T_ - NRS _Post-T_)/NRS _Pre-T_ × 100%. Pre-T, Pre-treatment; Post-T, Post-treatment; F, Frequency; EAM, External auditory meatus; Y, Yes; N, No.

### Case three: typical RHS with otalgia

The patient was a 67-year-old woman with a history of painful right EAM and ipsilateral facial paralysis without hearing loss and vertigo. She was diagnosed with typical RHS and was administered intravenous prednisolone (1 mg/kg per day), acyclovir (15 mg/kg per day), oral pregabalin (initial dosage of 75 mg three times a day and up to 150 mg three times a day), and mecobalamin (0.5 mg three times a day). Although the vesicular rash had disappeared 10 days later, the paroxysmal otalgia progressively worsened. Therefore, additional tramadol extended-release capsules (100 mg every 6 h) were administered orally, but this was ineffective. The patient was admitted to our pain clinic after 1 month for further treatment.

**Figure 6:**
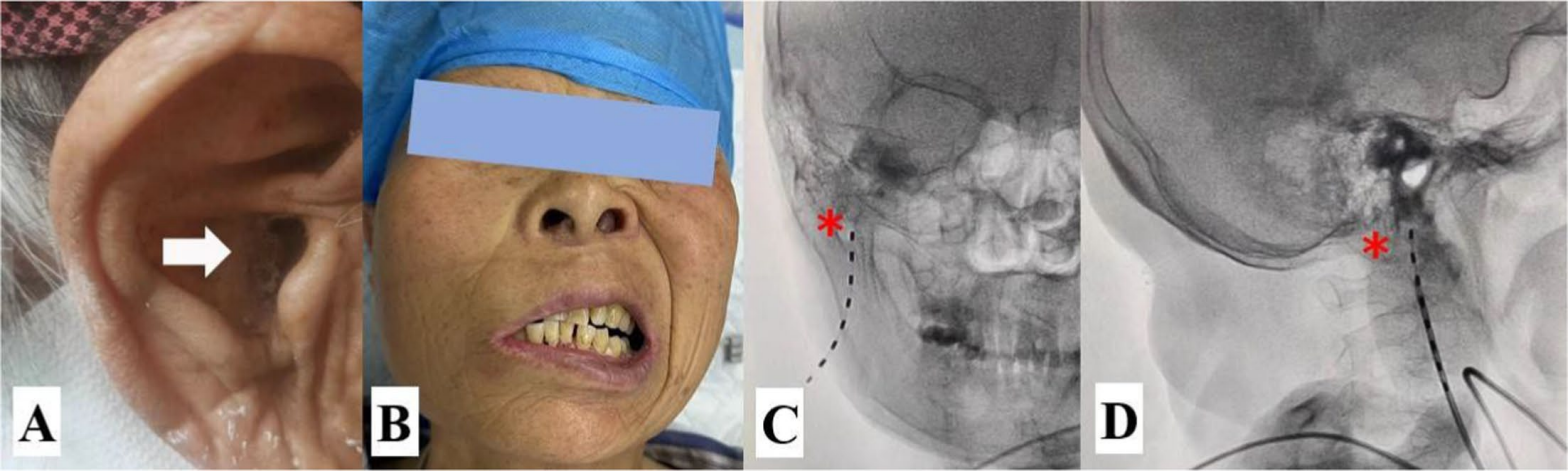
Clinical features and lead placement. **(A)** Cutaneous eschar in the EAM; **(B)** Facial palsy; **(C)** The lead tip was located medial to the MP in the anteroposterior view of the DSA. **(D)** The tip of the lead was located anterior to the MP in the lateral view of the DSA. The white arrow indicates the cutaneous eschar and pain location in the EAM, and the yellow asterisk indicates the MP.

She reported persistent pain with paroxysmal exacerbations of intensity. Her persistent pain was an “incessant tingling and itching” in the EAM with an intensity of 4/10 on the NRS, but her sharp and shooting otalgia was 8/10 on an NRS, lasted approximately 3–5 min, and occurred more than 10 times a day with indefinite interval time. Physical examination revealed a cutaneous eschar in the EAM and facial paralysis on the right side (Figures 6A,B). Routine blood and urine tests, magnetic resonance imaging of the brain, computed tomography, and electrocardiography findings were normal. She expressed heightened fear and anxiety about the possibility of experiencing next attack.

According to her medical history of symptoms and physical signs, she was diagnosed with typical RHS with severe otalgia. The FNS was recommended, and the risks, benefits, and alternatives of the procedure were discussed with the patient. After obtaining informed consent, the procedure was performed in accordance the pilot protocol (Figures 6C,D).

The t-FNS significantly relieved both the persistent pain and the breakthrough pain in EAM, especially the breakthrough pain. The patient’s persistent otalgia decreased significantly from moderate to mild after treatment for 5 consecutive days, and the mild persistent pain completely resolved at the 2-month follow-up. The severe pain attack decreased to moderate pain immediately after the start of treatment and disappeared, and the breakthrough pain had completely disappeared for 5 consecutive days after treatment. The frequency of seizures decreased from more than 10 times a day before treatment to 4 times on the first day, twice on the third and fifth day, and finally disappeared. The duration of the individual seizures also decreased from 3 to 5 min before treatment to 1 min, 20 s, and 5 s on the first, third, and fifth day, respectively (Table 3).

**Table 3:** The efficacy of transcutaneous facial nerve stimulation (t-FNS).

Nature	Standard		Pre-T	Post-T													
				1D	3D	5D	7D	9D	11D	13D	15D	1 M	2 M	3 M	6 M	9 M	1Y
Persistent Pain	Location (Y/N)	EAM	Y	Y	Y	Y	Y	Y	Y	Y	Y	Y	N	N	N	N	N
	VRS		2	2	2	1	1	1	1	1	1	1	0	0	0	0	0
	PRS			2	2	3	3	3	3	3	3	3	4	4	4	4	4
	NRS		4	2	2	1	1	1	1	1	1	1	0	0	0	0	0
	PRR (%)			50	50	75	75	75	75	75	75	75	100	100	100	100	100
Breakthrough Pain	Location (Y/N)	EAM	Y	Y	Y	Y	N	N	N	N	N	N	N	N	N	N	N
	F (per-day)		>10	4	2	2	0	0	0	0	0	0	0	0	0	0	0
	Duration		3-5 min	1 min	20 Sec	5 Sec											
	VRS		4	3	2	1	0	0	0	0	0	0	0	0	0	0	0
	PRS			2	3	3	4	4	4	4	4	4	4	4	4	4	4
	NRS		8	5	4	2	0	0	0	0	0	0	0	0	0	0	0
	PRR (%)			37.5	50	75	100	100	100	100	100	100	100	100	100	100	100
Allodynia	Y/N		Y	Y	Y	Y	Y	Y	Y	Y	Y	Y	N	N	N	N	

Patients reported their pain intensity using the Numerical Rating Scale (NRS) (‘0’ means no pain and ‘10’ means unbearable pain) and the Verbal Rating Scale (VRS) (no pain = 0; mild pain = 1; moderate pain = 2; and severe = 3, intense pain = 4). Patients rate the treatment using the using the Pain Relief Scale (PRS) (none = 0, a little = 1, some = 2, a lot = 3, complete = 4). In addition, the Pain Relief Ratio (PRR) was calculated as (NRS _Pre-T_ - NRS _Post-T_)/NRS _Pre-T_ × 100%. Pre-T, Pre-treatment; Post-T, Post-treatment; F, Frequency; EAM, External auditory meatus (EAM); Y, Yes; N, No.

## Discussion

Among the three cases, two atypical cases of RHS with severe neuralgia in unusually extensive dermatomes without facial paralysis and one typical case of RHS with severe otalgia and facial paralysis. Typical RHS is known to cause otalgia through its effects on the facial nerve (CN VII). However, atypical RHS affects multiple cranial nerves, particularly V, VII, VIII, and XII, as well as the cervical nerves, and often causes severe neuralgia in unusually widespread facial and cervical dermatomes (Sun et al., 2011; Xanthopoulos et al., 2002). Although the first case experienced unusually widespread pain, it disappeared immediately after the facial nerve was blocked. Therefore, her widespread pain in the frontal-temporal, mandibular, pharyngeal, upper cervical, and occipital regions was caused by the breakthrough of the facial nerve postherpetic neuralgia. The second case also experienced unusually widespread pain, but he suffered from a rash in the external auditory canal and frontal-temporal region, and his pain in the frontal-temporal region decreased significantly after blockage of the supraorbital nerve. His widespread pain in the frontal-temporal, infraorbital, mandibular, pharyngeal, upper cervical, and occipital regions also disappeared immediately after blocking the facial nerve. Thus, case 2 was diagnosed as atypical RHS with CN V involvement. The third case was diagnosed as a typical RHS with severe otalgia based on her clinical features.

The mechanism of RHS neuralgia is primarily related to polycranial neuritis, in which both sensory and motor nerves are significantly damaged. Some patients, especially those with underlying conditions such as diabetes, severe infections that further exacerbate the nerve damage and increase the neuralgic pain, making them resistant to conventional treatments (Sun et al., 2011). The pathophysiology underlying the intractable pain appears to stem from viral-induced nerve inflammation and subsequent neuronal degeneration. This neuronal damage disrupts normal sensory signal transmission, resulting in persistent and severe pain sensations even after the primary infection subsides (Alicandri-Ciufelli et al., 2012). The interplay between neuronal inflammation, degeneration, and persistent sensitization of pain pathways contributes to the chronicity and intractability of neuralgia associated with RHS (Sun et al., 2011).

Although there are various treatments for postherpetic neuralgia (PHN), spinal cord stimulation (SCS) is an established treatment option for patients with pharmacologically resistant PHN (Isagulyan et al., 2023). Recently, some studies on peripheral nerve stimulation (PNS) have actively investigated and increasingly used in clinical practice. This therapeutic approach uses electrical stimulation of the peripheral nerves to relieve pain and has shown promise in the treatment of PHN. Yakovlev and Peterson (2007) detailed the PNS in the right subscapular and right paraspinal areas of the upper thoracic region in a case with intractable PHN in which conventional treatment failed to provide improvement, highlighting success in the treatment of PHN (Yakovlev and Peterson, 2007). Several case studies have further illustrated the successful outcomes of PNS for the treatment of herpetic trigeminal neuralgia (Wan and Song, 2021; Liu et al., 2021; Han et al., 2020). In the current report, the first patient experienced about 20% pain relief for approximately 4 h after PRF of the facial nerve was performed. Therefore, t-FNS was recommended to relieve her PHN. Our results align with prior research on neuromodulation for neuropathic pain, which demonstrated varying degrees of pain relief in refractory cases. Compared to transcutaneous electrical nerve stimulation (TENS) and repetitive transcranial magnetic stimulation (rTMS), t-FNS has proven to be a reliable method for RHS neuralgia with fewer procedural risks.

In terms of efficacy, t-FNS showed good therapeutic results for different pain patterns. At the end of treatment, the paroxysmal pain in three patients and persistent pain in two patients completely disappeared. Only one patient experienced long-term mild pain in the distribution area of a branch of the trigeminal nerve, which continued to decrease over time. In terms of safety, this technique is performed under the combined guidance of ultrasound and DSA. Ultrasound monitoring can prevent vascular damage, whereas DSA guidance enables precise implantation of the electrodes in the target area. Anesthesia is provided through intravenous sedation and local anesthesia. Intravenous sedation not only alleviates the patient’s anxiety during the procedure but also allows for timely reporting of complaints. This proactive communication can help prevent the occurrence of complications such as nerve and vascular injuries. A local infiltration of 10 mL of 0.5% lidocaine is injected into the tissue from the skin to the stylomastoid foramen, which not only provides effective pain relief but also dilates the puncture channel. Thus, the risk of nerve and vascular injury was further reduced during the procedure. No procedure-related adverse events were observed during follow-up. All patients tolerated the intervention well, with no reports of infection, bleeding, or unintended nerve stimulation effects. Although no complications were observed in this study, potential risks such as transient discomfort at the stimulation site, device-related irritation, or inadequate response should be considered in future research.

Despite its promising results, this study has several limitations. First, as a retrospective case series with a small sample size, the findings may not be generalized to a broader patient population. Selection bias may also be present because only patients who received t-FNS were included. Additionally, the lack of a control group prevents direct comparisons with other treatment modalities. Future research should focus on optimizing stimulation parameters (e.g., frequency, intensity, and duration) to maximize clinical efficacy. Finally, prospective trials with longer follow-up periods are essential to assess the durability of treatment effects and to identify predictors of long-term success.

## Conclusion

t-FNS provides immediate and long-term pain relief for patients suffering RHS neuralgia refractory to conventional therapy. The procedure was performed under ultrasound guidance in combination with DSA, effectively avoiding procedure-related complications. These findings suggest that t-FNS may serve as a promising minimally invasive neuromodulation option for patients who have exhausted conventional treatments. However, given the limitations of this case series, additional well-designed controlled trials are necessary to confirm its efficacy, establish optimal patient selection criteria, and standardize treatment protocols for broader clinical application.

## Data Availability

The original contributions presented in the study are included in the article/supplementary material, further inquiries can be directed to the corresponding author.
